# The role of innate lymphoid cells (ILCs) in mental health

**DOI:** 10.1007/s44192-022-00006-1

**Published:** 2022-02-07

**Authors:** Tatiana Barichello

**Affiliations:** 1 Faillace Department of Psychiatry and Behavioral Sciences, McGovern Medical School, The University of Texas Health Science Center at Houston (UTHealth), Houston, TX USA; 2 Laboratório de Fisiopatologia Experimental, Programa de Pós-Graduação Em Ciências da Saúde, Universidade Do Extremo Sul Catarinense (UNESC), Criciúma, SC Brazil

**Keywords:** Innate lymphoid cells, ILCs, Cytokines, Gut, Neuroinflammation, Mental health

## Abstract

One hundred and thirty years after lymphoid and myeloid cells were discovered, in 2008, the researchers presented to the scientific community the population of innate lymphoid cells (ILCs) identified in humans and mice. Human ILC subsets were first identified in secondary lymphoid tissues and subsequently reported in the intestine, lung, liver, skin, and meninges. ILCs (ILC1, ILC2, ILC3, and ILCreg) subgroups present plastic properties concerning cytokines, chemokines, and other mediators present in the microenvironment. ILC1s were characterized by their ability to produce interferon (IFN)-γ. ILC2s have a function in innate and adaptive type 2 inflammation by producing effector cytokines such as interleukin (IL)-5 and IL-13. Meningeal ILC2s were activated in an IL-33-dependent mechanism releasing type-2 cytokines and demonstrating that ILC2s proliferate in reaction to IL-33 activation. ILC3s have been discovered as a significant contribution to the homeostasis of the gut barrier and as a source of IL-22. IL-22 presents a pleiotropic activity reinforcing the gut barrier immunity by stimulating anti-microbial peptide synthesis and promoting microbial regulation. Additionally, ILCs can have a pathogenic or protective effect on many disorders, and further research is needed to determine what elements influence the nature of their actions in diverse situations. The narrative review summarizes the role of the ILCs in mental health.

## Introduction

Innate lymphoid cells (ILCs) are the most recently reported immune cells, identified as a novel type of non-T and non-B lymphocytes. The newly discovered ILC1s, ILC2s, and ILC3s, which generate cytokines similarly with T helper (Th)-1, Th-2, and Th-17/Th-22 cells types, respectively, were incorporated in a related cell subset that also includes natural killer (NK) and lymphoid tissue inducer (LTi) cells [1–3]. ILCs play a vital role in the regulation of immunity, inflammation, and barrier homeostasis by producing cytokines in response to tissue-derived signals, damage-associated molecular patterns (DAMPs), pathogen-associated molecular patterns (PAMPs), microbe-associated molecular patterns (MAMPs), or other environmental stimuli [2].

**Fig. 1:**
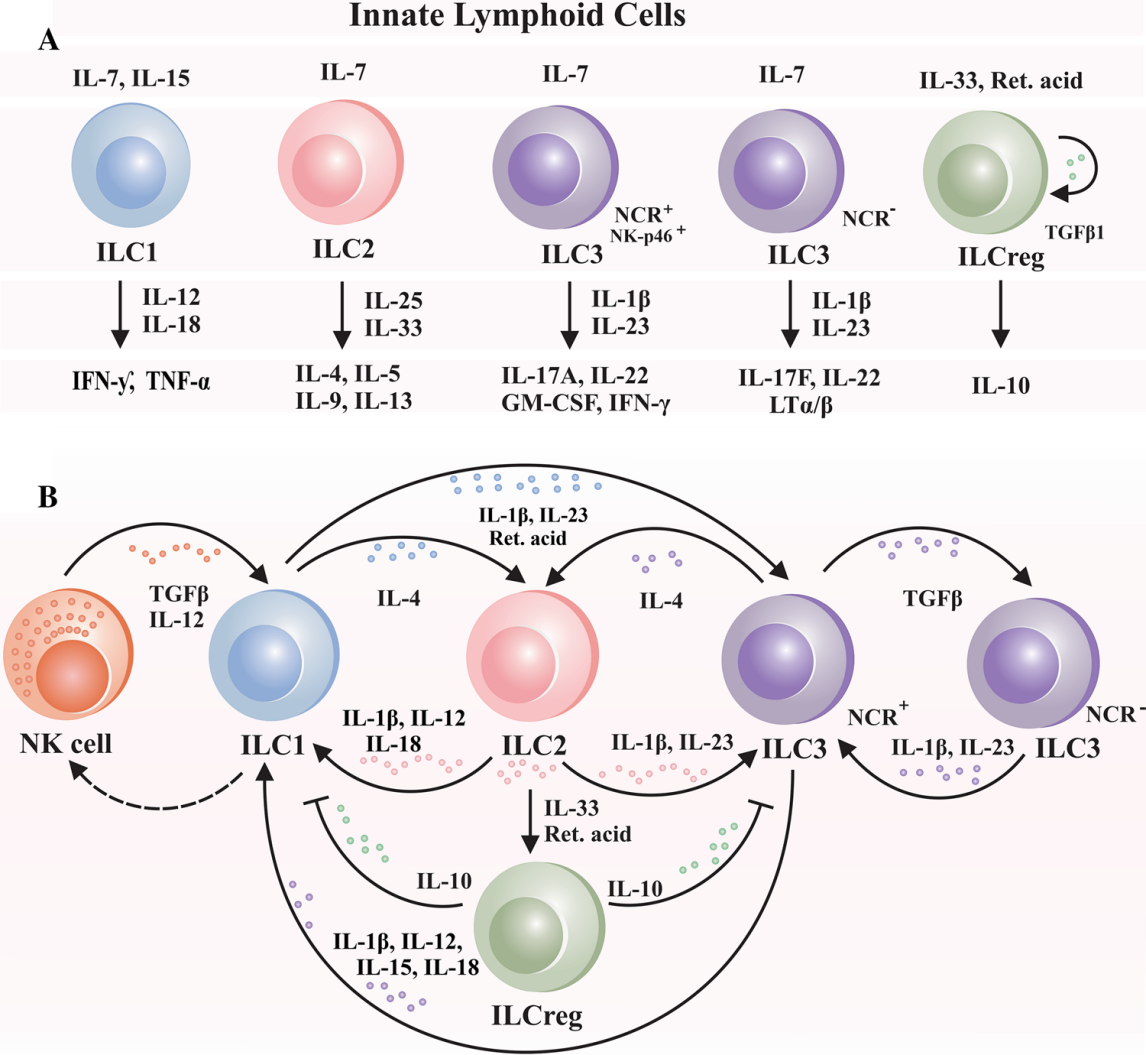
Innate lymphoid cells (ILCs). **A** Different cytokines and chemokines activate ILCs, and following activation, ILCs release cytokines and chemokines into their surroundings. **B** The ILCs can modify their outcome, as well as their function and phenotype, in response to environmental stimuli

Human ILC subsets were first identified in secondary lymphoid tissues and subsequently reported in the intestine, lung, liver, and skin. ILC1s are characterized by their capacity to produce interferon (IFN)-γ dependent on the transcription factor T-box expressed in T cells (T-bet) that directly activates IFN-gene production. ILC2s are GATA-binding protein-3 (GATA3) transcription factor-dependent and may generate interleukin (IL)-4, IL-5, IL-9, and IL-13. ILC3s, natural cytotoxicity receptor (NCR)^+^, are dependent on the retinoic acid receptor-related orphan receptor-t (RORt) for development and function in order to produce IL-17A, IL-22, GM-CSF, and IFN-ƴ. ILC3s, NCR^−^, release IL-17F, IL-22, and LTα/β [4]. ILC2_10_, an ILC-regulatory cell that expresses the inhibitor of differentiation/DNA binding (ID)-3 transcription factor, has recently been discovered to have regulatory characteristics due to its IL-10 activation in response to IL-33 retinoic acid stimulation [4, 5]. The ILCs have the ability to communicate with the surrounding microenvironment and present biological roles due to their cellular plasticity [4]. Each ILCs responds to distinct stimuli, IL-7, IL-12, IL-15, and IL-18 trigger ILC1s phenotype; IL-1β, IL-7, IL-33, and retinoic acid trigger ILC2s phenotype; and IL-1β, IL-7, and IL-23 trigger ILC3s phenotype. ILC2s and ILC3s transdifferentiate into ILC1s in response to IL-1β, IL-15, IL-12, whereas IL-1β and IL-23 can drive the plasticity of ILC1s and ILC2s towards ILC3s [4, 6]. Figure 1 summarizes the ILCs subgroups and their plasticity properties concerning cytokines and chemokines in their microenvironment. ILC2s and ILC3s have antigen presentation activity mediated by a major histocompatibility complex (MHC Class II), allowing them to interact directly with CD4^+^ T cells [7]. However, ILCs can have a pathological or protective effect on different diseases, and there is still necessary to investigate and understand what factors influence the nature of their functions in different environments [8, 9]. This narrative review recapitulates the role of the ILCs in mental health.

### Circulating ILCs

Pluripotent hematopoietic stem cells also differentiate into the common lymphoid progenitor (CLP) cells. Murine ILC cells originate first in the fetal liver and later in the adult bone marrow from CLPs. However, CLP in the human bloodstream has the ability to produce all ILCs, demonstrated both in vitro and in vivo studies [10]. The expression of hallmark transcription factor genes associated with the distinct ILC subsets is lacking in this cell type when they are released into the circulatory system after being produced in the bone marrow [11]. ILCs circulate like naïve immune cells, and the final step proposed is in lymphoid and non-lymphoid organs and on the body's mucosal surfaces [11]. A cluster of differentiation (CD127^+^) phenotype, which includes ILCs and NK subset of cells, was expressed by around 0.01 to 0.1 percent of circulating lymphocytes in healthy individuals. The vast majority of CD127^+^-ILCs identified in peripheral blood constitute ILCs of type-2 [12]. An investigation of the frequency and distribution of circulating ILCs populations in 89 healthy adult volunteers revealed that iILCs are present at meager rates, accounting for 0.034 percent on average. In contrast to the previous work, the distribution of ILC subsets in peripheral blood revealed a high proportion of ILC1s and a reduced proportion of ILC2s and ILC3s [13].

### The role of ILCs in the gut

The scientific community has recently investigated the involvement of the microbiota-gut-brain axis is in mental health. The bi-directional communication between the gut and brain occurs through the vagus nerve, immune system, enteroendocrine system, neurotransmitters, short-chain fatty acids (SCFAs), aryl hydrocarbon receptors (AHRs), bile acids, and the hypothalamic–pituitary–adrenal (HPA) axis [14, 15]. The most notable SCFAs produced by bacteria carbohydrate fermentation are acetate, propionate, and butyrate, which are involved in intestinal homeostasis, circadian rhythm, neuroimmune function, and behavior [16]. The SCFAs sense receptors on the ILCs in the gut, modulating the immune system and triggering the homeostasis, demonstrating that the gut microbiota is required for the ILCs to produce IL-22 [17]. IL-22 is a central regulator of the intestinal barrier that decreases gut permeability while increasing mucus production and anti-microbial peptides [18]. Several neuropsychiatry disorders are associated with gut permeability and bacterial translocation to the bloodstream, including Alzheimer's disease [19], Parkinson's disease [20], autism spectrum disorders [21], and schizophrenia [22, 23] among others [15, 16]. In the gut, ILC3s have been identified as a prominent contributor to the homeostasis of the barrier immunity. ILC3s produce the cytokine IL-22 in response to inflammatory stimuli and other pro-inflammatory cytokines and chemokines [24]. Then, (STAT)-3 phosphorylation increases by IL-22, as was IL-22-mediate epithelial regeneration in the gut [25, 26]. In addition, SCFAs increase IL-22 production by ILCs via G-protein receptor 41 (GPR41) and inhibit histone deacetylase (HDAC). SCFAs also stimulate IL-22 production via increasing the expression of the aryl hydrocarbon receptor (AHR) and the hypoxia-inducible factor 1 (HIF1) [27]. AHR is common in epithelial gut tissue and, when activated, improves intestinal epithelial barrier function and regulates immune responses. In a clinical study, psychological stress decreased IL-22 levels in the serum, demonstrating a reduced IL-22-induced protective immunity in the gut [28]. In another study, IL-22 administration induced the intestinal epithelial cells to increase tight junction protein expression in Crohn's disease patients; also, anti-TNF therapy increased IL-22 production in CD4^+^ T cells. Patients with inflammatory bowel disease (IBD) are at increased risk for mental health issues, including depression [29]. The IBD patients present an increase of the ILC1s and the ILC3s in the gut increasing the production of IFN-ƴ [30]. Currently, the literature has emphasized how the imbalance of gut homeostasis affects mental health [15, 16].

### The role of ILCs in the central nervous system

The SCFAs can be delivered from the gut to the circulatory system, crossing the BBB into the brain. In the brain, SCFAs bind to the AHR on the microglia and astrocyte cells blocking nuclear factor kappa-B (NF-κB) by activating the suppressor of cytokine signaling-2 (SOCS2). AHR activation decreases inflammation, neurotoxicity, and immune cell recruitment by increasing the transforming growth factor-alpha (TGF-α) and decreasing the vascular endothelial growth factor B (VEGFB) [31]. In addition, a network-based ranking algorithm study demonstrated that SCFAs are mechanistically involved in microglia-mediated microbiota-gut-brain axis connections in Alzheimer's disease at genetic, functional, and phenotypic levels [32]. Also, the amyloid SUVR uptake was negatively correlated with butyrate blood levels in Alzheimer's disease patients [33].

In the CNS, the meningeal lymphatic vasculature lines the dural sinuses. These tissues exhibit all of the morphological features of lymphatic endothelial cells that can transport fluid and, most importantly, connect with immune cells present in the cerebrospinal fluid (CSF) [34]. Similarly, the meninges act as a CSF barrier, giving CNS-resident ILCs an advantageous anatomical site to act as cerebral immune gatekeepers, conveying information from the brain to the immune system [35]. ILC2s were found in mouse meninges and are most prominent near the dural sinuses. Meningeal ILC2s were activated in an IL-33-dependent mechanism after spinal cord injury (SCI), releasing type-2 cytokines and demonstrating that ILC2s proliferate in response to IL-33 activation [36]. Constitutively synthesized intracellular IL-33 helps maintain barrier function by regulating gene expression as a nuclear protein in healthy conditions. Nuclear IL-33, on the other hand, serves as a stored alarmin, which is triggered when barriers are disrupted. As the adaptive immune response is generated, extracellular IL-33 coordinates immunological defense and repair pathways while also triggering the development of Th-cells [37].

In Alzheimer's pathology, IL-33 was a crucial cytokine for aquaporin-4 (AQP-4) expression in the astrocytes endfeet, and to the glymphatic system eliminates the abnormal protein tau accumulation in the mouse brain [38]. IL-33 improved Alzheimer's disease-like pathology modulating IL-1β, IL-6, and NLR family pyrin domain containing 3 (NLRP3) genes in the cortices of APP/PS1 mice and decreasing cognitive impairment [39]. In another study, ILC2 in the brain was quantitatively reduced and functionally inefficient in both sexes' triple-transgenic Alzheimer's mouse models (3xTg-AD). The remnant ILC2 could not produce the type-2 cytokine IL-5; however, it developed the ability to express a variety of pro-inflammatory genes, including Granzyme-A (*Gzma*), a cytotoxic molecule. However, the IL-5 administration improved spatial recognition and learning memory in 3xTg-AD mice [40]. ILC2 has a pleiotropic property demonstrating multiple roles in distinct cell types; in a preclinical experiment, male-specific protection was provided by the mast cell and ILC2 connection in autoimmune encephalomyelitis (EAE) [41]. Also, decreased levels of IL-33 restricted ILC2 activation and promoted susceptibility in a female transgenic mice model of EAE to develop multiple sclerosis [8]. Overexpression of IL-33 in cellular models resulted in a selective reduction in the production of the Aβ_1-40_ peptides, a significant component of cerebral amyloid angiopathy (CAA) [42]. ILC-deficient mice displayed increased microglial reactivity and exacerbated neuroinflammatory responses in experimental models of EAE and skin/brain inflammation induced by imiquimod drug administration [43]. In an experimental model of inflammation induced by lipopolysaccharide (LPS), IL-13 controled brain inflammation by promoting the death of activated microglia in vivo, increasing neuronal survival cells [44]. IL-13 reduced neuroinflammation and promoted recovery after a mouse model of experimental traumatic brain injury [45]. Moreover, in an experimental model of ischemic stroke, peripheral administration of IL-13 reduced lesion volume, induced anti-inflammatory microglial and macrophage phenotypes, providing a neuroprotection [46]. In addition, the anti-inflammatory cytokine, IL-10, decreased the severity of inflammation and the blood–brain barrier permeability in an experimental model of severe acute pancreatitis [47]. In a clinical study, increased IL-1β and IL-18 production, along with pro-inflammatory cytokines, were connected to a significant decrease in IL-33 plasma of autism spectrum disorder patients [48]. In another study, patients with amnestic mild cognitive impairment or Alzheimer's disease who lacked IL-33 expression showed severe cognitive impairment, whereas patients who presented the IL-33 expression maintained their cognitive performance [49]. IL-33 expression was shown to be lower in the brains of Alzheimer's disease patients when compared to controls, and additional genetic investigation revealed three variants within the IL-33 gene, indicating a protective haplotype linked with Alzheimer's disease risk in non-APOE e4 carriers patients. These polymorphisms were likewise linked to lower CAA levels in the brains of non-APOE e4 AD patients [42]. Increased inflammatory biomarkers such as tumor necrosis factor-alpha (TNF)-α, IL-6, cyclooxygenase (COX)-2, and arachidonic acid (AA) were identified in bipolar disorder patients compared to healthy controls without mental illnesses; however, IL-10 and IL-33 levels remained unchanged [50]. On the contrary, another study found that IL-33 levels in the blood were higher in bipolar disorder patients than healthy controls [51]. The effect of IL-33 activation or inhibition on different diseases might be attributed to its biding effect on IL-33 receptors, which are found on mast cells, endothelial cells, microglia, and astrocytes in the brain. In the bloodstream, increased levels of IL-33, a nuclear-associated cytokine, can be connected with cellular apoptosis and necrosis. Also, Th-2 lymphocytes, macrophages, dendritic cells, CD8^+^ T cells, B cells, and certain granulocytes, including basophils and eosinophils, have been found to express IL-33 receptors [52], highlighting the importance of determining the mechanisms behind the IL-33 source. Furthermore, the connection between ILC2 and IL-33 must be investigated since ILC2's pleiotropic characteristics may be at the root of various neuropsychiatric disorders.

**Fig. 2:**
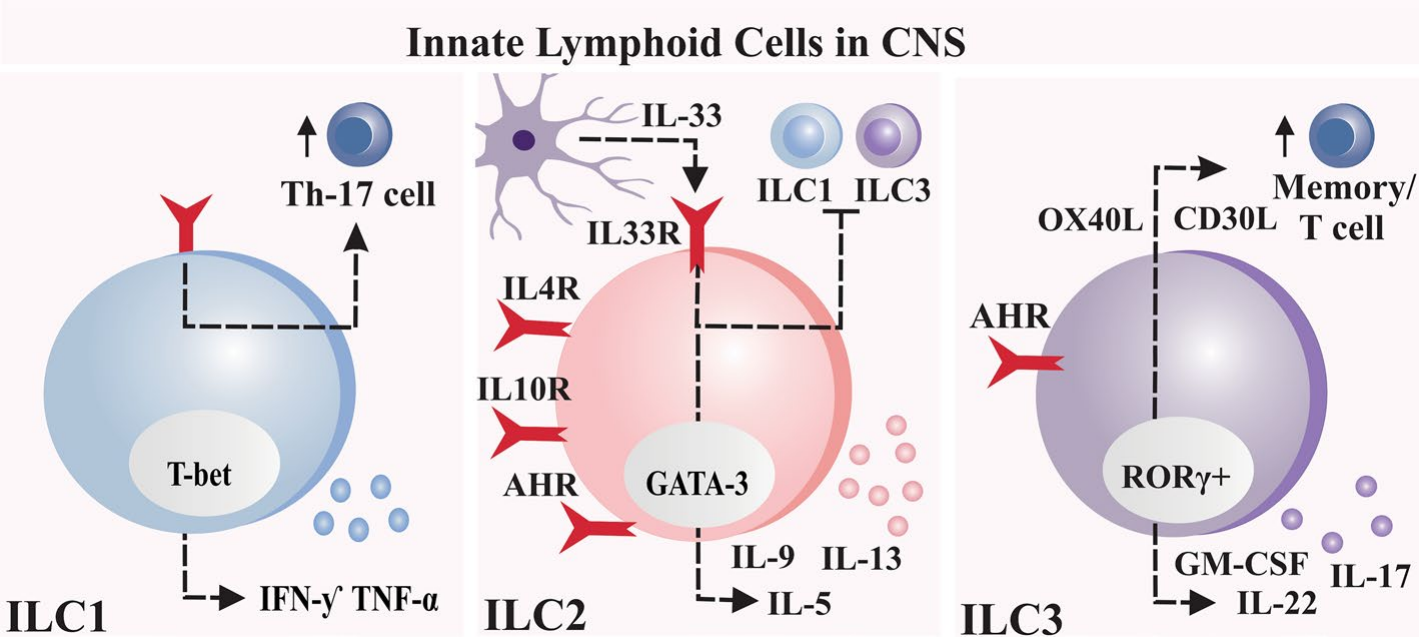
Innate lymphoid cells (ILCs) in the CNS. ILC1s facilitate the direct infiltration of Th-17 cells into the brain parenchyma and spinal cord, also release IFN-ƴ and TNF-α. IL-33, a cytokine released by healthy glial cells, activate ILC2s by blocking the ILC1 and ILC3 proliferation. ILC3s can release OX40L and CD30L molecules increasing the proliferation and survival of the memory T cells in the brain parenchyma and spinal cord

The ILC1s and ILC3s are normal meningeal and CNS parenchymal inhabitants. ILC1s facilitate the direct infiltration of Th-17 cells that mediate pro-inflammatory cytokines into the brain parenchyma and spinal cord, consequently contributing to the propagation of neuroimmune response to CNS injuries [53]. T cells that produce IL-17 are vital players in Parkinson's disease, which is connected with neurodegeneration [54]. ILC3s can release OX40L and CD30L molecules, a binding ligand, which increases the proliferation and survival of the memory T cells in the brain parenchyma and spinal cord [55]. In an EAE mouse model, ILC3s cells accumulated and exhibited disease-induced activation in the meninges of the animals [55]. The ILC1s and ILC3s in the brain are unknown and have only been described in acute brain damage, autoimmune diseases, inflammation, and infection. Only ILC2s have been explored in the context of neurodegeneration and mental health status [56]. For more details about the ILCs' function in the CNS, see Fig. 2.

## Conclusion

ILCs are necessary to be investigated since the results generated may lead to new and more effective treatments, mainly because the meninges are located outside of the BBB and, therefore, more easily targeted. ILC1s and ILC3s are essential targets to be investigated in order to treat diseases associated with infection and inflammation, including traumatic brain injury, autoimmune encephalomyelitis, sepsis, meningitis, among others. Otherwise, preclinical and clinical pieces of evidence have demonstrated the involvement of meningeal populations of type-2 innate lymphoid cells, ILC2s, in neuroinflammation and neurodegenerative diseases. Targeting the ILC2s may be a new avenue to investigate and possibly be a target to treat and improve mental health.
